# Cervical cancer screening in a low‐resource setting: a pilot study on an HPV‐based screen‐and‐treat approach

**DOI:** 10.1002/cam4.1089

**Published:** 2017-06-04

**Authors:** Margot Kunckler, Fanny Schumacher, Bruno Kenfack, Rosa Catarino, Manuela Viviano, Eveline Tincho, Pierre‐Marie Tebeu, Liliane Temogne, Pierre Vassilakos, Patrick Petignat

**Affiliations:** ^1^ Gynecology Division Department of Obstetrics and Gynecology University Hospitals of Geneva Geneva Switzerland; ^2^ Department of Biomedical Sciences University of Dschang Dschang District Hospital Dschang Cameroon; ^3^ Faculty of Medicine and Biomedical Sciences Centre Hospitalier Universitaire (CHUY) Yaoundé Cameroon; ^4^ Geneva Foundation for Medical Education and Research Geneva Switzerland

**Keywords:** Cervical cancer, HPV, HPV testing, screen‐and‐treat, self‐sampling

## Abstract

Cervical cancer (CC) is the leading cause of cancer‐related death among women in sub‐Saharan Africa, primarily because of limited access to effective screening and preventive treatment. Our aim was to assess the feasibility of a human papillomavirus (HPV)‐based CC screen‐and‐treat approach in a low‐resource context. We recruited 1012 women aged 30–49 years through a CC screening campaign conducted in the District Hospital of Dschang, Cameroon. Participants performed HPV self‐sampling, which was tested for high‐risk HPV (HR‐HPV) DNA using the point‐of‐care Xpert HPV assay. All HPV‐positive women were invited for visual inspection with acetic acid and Lugol's iodine (VIA/VILI) to exclude CC or enable triage. A cervical sample for histological analysis was also collected. Women positive for HPV 16/18/45 and for other HR‐HPV with pathological VIA/VILI were selected to undergo treatment with thermocoagulation. The HPV prevalence in the study population was 18.5% (*n* = 187); of these cases, 20 (10.6%), 42 (22.3%) and 140 (74.9%) were positive for HPV16, HPV18/45 and other HR‐HPV types, respectively. Overall, 107/185 (57.8%) VIA/VILI examinations were classified as pathological and 78 (42.2%) as normal. Women positive for HPV16/18/45 were 4.2 times more likely to harbor cervical intraepithelial neoplasia grade 2 or worse (CIN2+) than those with other HPV types. The specificity of HPV 16/18/45 genotypes for detection of high‐grade lesions among HR‐HPV positive women was higher than that of VIA/VILI in all age groups. The sensitivity and specificity of VIA/VILI in detecting CIN2+ among HPV positive women were 80% and 44%, respectively. Overall, 110/121 screen‐positive women (90.9%) were eligible for, and were treated with, thermocoagulation. An HPV‐based screen‐and‐treat approach is feasible in a low‐resource context and may contribute to improving the effectiveness of CC prevention programs. Immediate thermocoagulation treatment for women who are HPV16‐ and/or HPV18/45‐positive is a practical approach for the treatment of CIN2+. The combination of HPV‐testing and VIA/VILI for CC screening might reduce overtreatment.

## Introduction

Cervical cancer (CC) is the second most common cancer in women worldwide, and is a leading cause of cancer death in women in low‐resource countries such as Cameroon, despite being largely avoidable. Although cytology‐based CC screening programs have successfully reduced CC incidence in high‐income countries, such dramatic reduction in incidence has not been observed in low‐ and medium‐income countries (LMIC) 1. The incidence of invasive CC in sub‐Saharan Africa is expected to increase in the near future because of limited access of the population to information and health care facilities, an absence of sustained prevention programs, and high HIV prevalence 2.

Recent studies have shown that HPV‐based screening has a greater sensitivity than conventional cytology‐based screening programs for the detection of precancerous lesions and CC 3, 4. Furthermore, HPV‐based screening allows the safe extension of the screening interval to at least 5 years 4. Compared with the more frequent screening visits required by cytology‐based programs, the use of a highly sensitive test once or twice in a lifetime is more effective and applicable in low‐resource settings 5. Recent evidence has demonstrated that the quality of self‐collected cervicovaginal samples is similar to that of samples obtained by physicians for the detection of cervical intraepithelial neoplasia grade 2 or worse (CIN2+) lesion 6. HPV self‐sampling is generally better accepted by women than gynecological examination and could potentially increase screening participation rates 7, 8. Moreover, it offers the potential to reduce medical costs and overcome shortages of qualified staff 9.

Recently, a point‐of‐care assay (Cepheid's GeneXpert^®^, hereafter referred as “Xpert”) has become available. This method uses real‐time PCR to detect HR‐HPV DNA and identify genotype (e.g., HPV16, 18/45). Xpert is a quick (1‐hour), non‐batch HPV assay that could facilitate same‐day screening and management strategies 10. The emergence of rapid testing presents new possibilities for HPV‐based CC screening, especially in developing countries 11. For example, the technology makes it possible to screen and treat women in a 1‐ or 2‐day session. This is particularly important considering the difficulty in recalling women for further management, which is a major cause of low program impact in LMIC 12. Loss to follow‐up in the developing world likely results from difficulties in obtaining and affording additional services such as transport and child care. Therefore, a screen‐and‐treat approach, by combining diagnosis with immediate treatment in a same‐day session, has the potential to increase program effectiveness and to facilitate efficient allocation of the available human and financial resources. The visual inspection method in the context of a screen‐and‐treat approach has already been evaluated for CC prevention in LMIC and appears to be both safe and efficient, which makes it applicable as a triaging method 13.

Our project aims to implement a primary CC screening program based on a same‐session visit, which includes diagnosis and immediate treatment, if needed, by thermocoagulation.

## Materials and Methods

### Population and setting

This study was conducted between July and October 2015 in the District Hospital of Dschang, Cameroon. The Dschang health district is a semirural area located in the Western Region of Cameroon with an estimated population of 250,000. A total of 1012 women were recruited as part of a CC screening campaign, through announcements made at the hospital and banners displayed in public areas. Ethical approval was obtained from the Cameroonian National Ethics Committee for Human Health Research and the Ethical Cantonal Board of Geneva, Switzerland (CER: 15–068). Women were included if they were aged 30–49 years, understood the study procedures and voluntarily agreed to participate by signing an informed consent form. Exclusion criteria were pregnancy*,* previous total hysterectomy and inability to comply with the study protocol. The trial is registered with the study ID ISRCTN99459678 in the ISRCTN registry.

### Sample collection and laboratory analysis

Socio‐demographic data and gynecological and obstetrical history were collected to evaluate the association of risk factors with HPV infection. After being informed about HPV infection and CC, the participants were invited to perform HPV self‐sampling. Dry cotton swabs were used for cervicovaginal sampling, and samples were immediately prepared for on‐site analysis. The swab was introduced in a tube containing 5 mL of NaCl medium and vortexed for 10 sec. After vortexing, 1 mL of each sample was transferred to the GeneXpert cartridge and run on a four‐module GeneXpert machine.

### HPV test

We used an Xpert HPV assay consisting of real time PCR that uses detection of a human reference gene (hydroxymethylbilane synthase, HMBS) and an internal Probe Check Control (PCC) as an internal assay control for specimen adequacy. The PCC was used to verify reagent rehydration, PCR tube filling in the cartridge, probe integrity and dye stability. The Xpert test included reagents for the simultaneous detection of 14 HR‐HPV genotypes (HPV16, 18, 31, 33, 35, 39, 45, 51, 52, 56, 58, 59, 66, and 68). The assay used multiple fluorescent channels for the detection of individual types of HPV, groups of HPV, and the human reference gene. Each fluorescent channel had specific cutoff parameters for target detection and validity. If a sufficient amount signal was detected for the human reference gene, the assay results were reported as an overall positive. Additionally HPV16, pooled HPV18/45 and pooled other HR‐HPV types detected by the assay were reported separately as positive or negative.

### Study design

**Figure 1:**
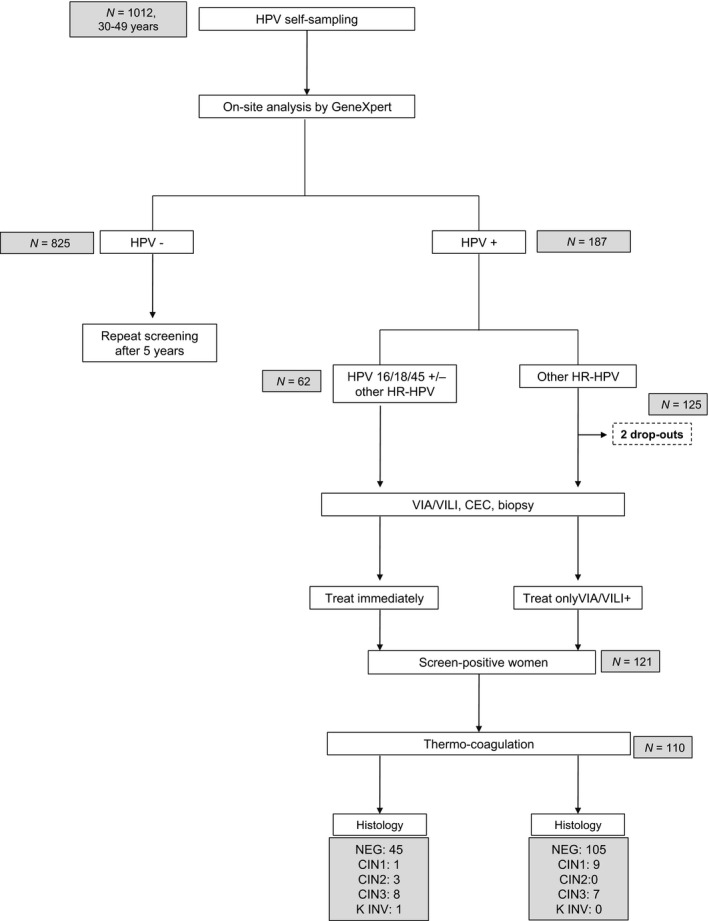
Study flowchart. *N*, number; HPV, human papillomavirus; VIA, visual inspection with acetic acid; VILI, visual inspection with Lugol's iodine; ECC, endocervical curettage; NEG, absence of cervical intraepithelial neoplasia at biopsy and/or ECC; CIN1, cervical intraepithelial; neoplasia grade 1; CIN2, cervical intra‐epithelial neoplasia grade 2; CIN3, cervical intraepithelial neoplasia grade 3.

Each HPV test was analyzed within 1 hour of sample collection and the results were immediately communicated to the participants. Depending on the women's HPV status, management was as follows: HPV‐negative women were reassured and advised to repeat the test in 5 years. HPV‐positive women (all HPV genotypes) with a normal VIA/VILI test underwent cervical 6 o'clock biopsy and endocervical curettage (ECC) to exclude CC, and for quality control. HPV‐positive women (all HPV genotypes) with an abnormal VIA/VILI test underwent biopsy of the abnormal area followed by thermo‐coagulation treatment. Additionally, women positive for HPV16 and/or HPV18/45 were immediately treated with thermo‐coagulation, regardless of the VIA/VILI outcome. A control visit at 1 month was arranged for all women treated with thermocoagulation in order to exclude the presence of posttreatment side effects, such as bleeding and infection. Follow‐up visits at 6 and 12 months were arranged for all HPV‐positive women in order to assess viral clearance. HPV‐positive women had three pictures of the cervix (native cervix, after VIA and after VILI) taken using a Smartphone (Samsung S5, Seoul, South Korea) for quality control. The study design is illustrated in the flowchart in Figure 1.

### Via/vili

VIA and VILI tests were performed by three trained local gynecologists. VIA consisted of application of a 3–5% acetic acid solution to the cervix. Appearance of acetowhite areas touching the squamocolumnar junction (SCJ) was used to help identify pathological areas of the cervix. VILI consisted of application of Lugol's iodine to the cervix. Appearance of a well‐defined, bright yellow area touching the SCJ indicated a suspicious lesion. VIA and VILI were conducted and the results interpreted according to the World Health Organization's recommendations 14.

### Cervical biopsy and ECC

We used biopsy forceps to perform cervical biopsy and an endocervical brush to perform ECC. Material collected was conserved in formalin‐based solution. An experienced pathologist, blind to the HPV and the VIA/VILI test results, conducted the histological analyses in Geneva. Histological analyses were conducted in accordance with Swiss standards and international recommendations.

### Thermo‐coagulation

Thermo‐coagulation involved application of a probe heated to 100°C to the cervix for 50 sec, to achieve an expected tissue destruction of 5–7 mm depth. An appropriately sized probe was selected based on the area of the pathological cervical tissue. Several applications were performed, if necessary, to cover the entire pathological area.

### Data management and statistical analysis

A personalized electronic medical chart including sociodemographic and medical information was created using the SecuTrial online database to collect and manage data. Sensitivity, specificity, positive predictive value and negative predictive value were calculated for the VIA/VILI, HPV16, HPV18/45 and HPV16/18/45 assays, using histopathology as the gold standard. A bivariate logistic regression was performed to identify factors associated with HPV prevalence. *P* < 0.05 was considered statistically significant. Analyses were performed for the whole study population and separately for women of different age groups. Relative risk of CIN2+ was calculated for the different HPV genotypes. Data analysis was conducted using Stata Statistical software Release 13 (StataCorp LP, College Station, TX).

## Results

### Participant characteristics

The socio‐demographic characteristics of the participants are shown in Table 1. The mean age of the participants was 39.6 ± 5.6 years. The majority of women (94.6%) had a partner. Overall, 805 (76.9%) participants had at least a secondary school degree. The majority of women did not use contraception (70.5%).

**Table 1:** Socio‐demographic and clinical characteristics of study participants (*n *=* *1012)

Variable	*N*	%
Total	1012	
Age (mean ± SD), y	39.6 ± 5.6	
Age groups, y		
30–34	232	23.0
35–39	257	25.4
40–44	280	27.7
≥45	241	23.9
Marital status		
Without a partner	55	5.5
With a partner	954	94.5
Education		
Unschooled	7	0.7
Primary education	223	22.2
Secondary education	618	61.5
Tertiary education	157	15.6
Work		
Employee	404	40.0
Independent	267	26.4
Housewife	259	25.6
Farmer	49	4.9
Other	31	3.1
Age at menarche (mean ± SD), y	14.8 ± 1.8	
Age of first sexual intercourse (mean ± SD), y	18.0 ± 2.5	
Number of sexual partners (mean ± SD)	3.7 ± 2.7	
Number of pregnancies, (mean ± SD)	5.5 ± 2.3	
Number of children (mean ± SD)	4.5 ± 1.9	
Age at first delivery (mean ± SD), y	21.8 ± 4.0	
Contraception		
Pill	18	1.8
Injectable	35	3.5
Intrauterine device	46	4.6
Condom	111	11.0
Other	87	8.6
None	709	70.5
Antecedents of cytological screening		
Yes	222	22.0
No	788	88.0
Family history of cervical cancer		
Yes	64	6.3
No	882	87.4
I don't know	64	6.3

*N*, number; SD, standard deviation; y, years.

### Disease prevalence

HPV prevalence was 18.5% (*n* = 187/1012). Eighteen (9.6%) samples were positive for HPV16, 29 (15.5%) were positive for HPV18/45, 125 (66.8%) were positive for other HR‐HPV types, 2 (1.1%) were co‐positive for HPV16 and other HR‐HPV types and 13 (7.0%) were co‐positive for HPV18/45 and other HR‐HPV types (Table 2). Nine (0.9%) tests were invalid and had to be repeated.

**Table 2:** Disease status

Variable	*N*	%
Overall HPV prevalence	187	18.5
HPV prevalence by type		
HPV 16	18	9.6
HPV 18/45	29	15.5
Other HR‐HPV	125	66.8
HPV 16+ other HR‐HPV	2	1.1
HPV 18/45+ other HR‐HPV	13	7.0
Disease status — VIA/VILI		
Pathological	107	57.8
Non pathological	78	42.2
Pathological VIA/VILI according to HPV type		
HPV 16	17	15.9
HPV 18/45	30	28.0
Other HR‐HPV	60	56.1
Histological diagnosis		
Negative	150	83.8
CIN1	10	5.6
CIN2+	19	10.6

*N*, number; HPV, human papillomavirus; CIN1, cervical intraepithelial neoplasia grade 1; CIN2+, cervical intraepithelial neoplasia grade 2 or worse.

A total of 185 (98.9%) HPV‐positive women underwent VIA/VILI examination. Overall, 107 (57.8%) VIA examinations were classified as pathological. A total of 121 (65.4%) women had a positive screen, of whom 110 (90.9%) were eligible to be treated with thermo‐coagulation. Histological analyses revealed 10 cases with (5.3% of women with an HPV positive screen) CIN1, 3 (1.6%) with CIN2, 15 (8.0%) with CIN3 and 1 (0.5%) with CC. Four histological samples were lost between the transportation and the laboratory analysis processes.

### Screen‐and‐treat process

Among 62 women positive for HPV 16/18/45, 61 (98.4%) underwent screening and treatment in the same 1‐day session. Among 126 women positive for other HR‐HPV types, 125 (99.2%) underwent VIA/VILI triage. Two women (1.1%) were lost between HPV testing and VIA/VILI triage. In total, 110 of the 121 women requiring treatment were treated in the same‐day session. Reasons for not being able to treat women with a positive screen included: no visualization of the SCJ (*n* = 1), presence of a lesion extending to more than 2 mm inside the cervix (*n* = 5), presence of a lesion suspicious for cancer (*n* = 3), failure of probe heating (*n* = 1) and undeclared pregnancy at the time of enrollment (*n* = 1). Women presenting with large pathological areas or with lesions suspicious for cancer were referred to LEEP or hysterectomy. Pregnant women were invited for a follow‐up visit after childbirth.

### Distribution of HPV infection and cervical precancer lesions according to age

HR‐HPV prevalence was highest among younger women (aged 30–40), and decreased in women aged 40 years and older. HPV 18/45 prevalence was highest among women aged 35–39 years (5.5%). HPV 16 prevalence increased with age, reaching a peak among women aged 40–45 (2.5%). CIN1 prevalence was higher among younger women. Most CIN3 lesions were observed in women older than 35 years.

### Prevalence of HPV infection by demographic and pathological characteristics

The likelihood of HR‐HPV infection decreased with age (OR = 0.96; 95% CI: 0.94–099; *P* = 0.015). Women who had had more than six sexual partners had 2.12 times the odds of presenting with a HPV infection (95% CI: 1.15–3.91; *P* = 0.016). Number of children was a protective factor for presenting with a HPV infection (OR = 0.87; 95% CI: 0.80–0.95; *P* = 0.001): women with more than two children had 0.66 times the odds to have an HR‐HPV infection as women with one or no children (95% CI: 0.44–0.99; *P* = 0.045).

### Performance of VIA/VILI and HPV 16 and HPV 18/45 genotyping for detection of high‐grade cervical lesions

Overall, the VIA/VILI positivity rate was higher when women were positive for HPV 16 (85.0%). The VIA/VILI positivity rate was 73.2% when women were positive for HPV 18/45, and 77.1% when women were positive for either HPV 16 or HPV 18/45 or both. The VIA/VILI positivity rate was lower (50.4%) in women positive for other HR‐HPV types. Performance of VIA/VILI and HPV genotyping for detection of high‐grade cervical lesions is presented in Table 3. The specificity of HPV 16/18/45 genotypes for detection of high‐grade lesions among HR‐HPV positive women was higher than that of VIA/VILI in all age groups. For women aged 30–34 years, the sensitivity of HPV 16 results was comparable with that of VIA/VILI results (66.7%). Among all HPV‐positive women, 19 (10.2%) had CIN2+ lesions. We found CIN2+ lesions in 7 HPV 16‐positive women (35%), 5 in HPV 18/45‐positive women (12%) and 10 in women positive for other HR‐HPV types (7.1%). The specificity of HPV 16 for detection of CIN2+ lesions was 91.9% (95% CI: 86.4–95.2).

**Table 3:** Age‐stratified performance of VIA/VILI and HPV 16/18/45 genotyping for detection of high‐grade cervical lesions among the 185 HR‐HPV‐positive women who underwent VIA/VILI

		Sensitivity	Specificity	PPV	NPV
Lesion	[*n* lesions]	% (95 % CI)	% (95 % CI)	% (95 % CI)	% (95 % CI)
Disease threshold CIN2+ for women 30–39 years old	[10]				
VIA/VILI		80.0 (0.34–96.3)	44.7 (34.8–55.0)	13.3 (6.7–24.9)	95.5 (82.7–98.9)
HPV 16(+)		30.0 (7.6–69.0)	94.7 (87.7–87.8)	37.5 (8.6–79.3)	92.7 (85.3–96.5)
HPV 18/45(+)		30.0 (7.6–69.0)	79.8 (70.3–86.8)	13.6 (4.0–37.2)	91.5 (82.9–95.9)
HPV 16/18/45(+)		60.0 (24.3–87.5)	74.5 (64.5–82.4)	20.0 (8.8–39.2)	94.6 (86.2–98.0)
Disease threshold CIN2+ for women ≥40 years old	[9]				
VIA/VILI		88,9 (37.4–99.1)	47.9 (33.8–62.3)	19.4 (8.5–38.1)	96.9 (79.1–99.6)
HPV 16(+)		44.4 (13.4–80.4)	87.8 (77.2–93.9)	33.3 (10.9–67.1)	92.1 (81.9–96.7)
HPV 18/45(+)		42.9 (9.1–85.0)	78.8 (67.0–87.2)	12.5 (2.6–43.0)	88.1 (76.7–94.4)
HPV 16/18/45(+)		66.7 (26.1–91.9)	66.7 (54.1–77.2)	21.4 (9.4–41.7)	93.6 (81.3–98.0)
Disease threshold CIN2+ for women all ages	[19]				
VIA/VILI		84.2 (57.8–95.3)	45.6 (38.0–53.5)	15.6 (9.7–24.0)	96.1 (88.2–98.8)
HPV 16(+)		36.8 (17.3–62.0)	91.9 (86.4–95.2)	35.0 (16.4–59.6)	92.5 (87.1–95.7)
HPV 18/45(+)		26.3 (10.4–52.4)	79.4 (72.3–85.0)	13.2 (5.4–28.9)	90.1 (83.8–94.1)
HPV 16/18/45(+)		63.2 (38.0–82.7)	71.3 (63.7–77.8)	20.7 (11.9–33.4)	94.2 (88.2–97.2)

CIN2+, cervical intraepithelial neoplasia grade 2 or worse; PPV, positive predictive value; NPV, negative predictive value.

### Relative risk of high‐grade cervical disease according to age groups

Women aged 30–34 years who were positive for HPV 16 had 90 times greater risk of having a CIN2+ lesion than those who were negative for HPV1 6 (*P* = 0.005). Among women of all ages, those who were positive for HPV 16 and/or HPV 18/45 had 4.2 times greater risk of having a CIN2+ than those who were positive for other HR‐HPV types (*P* = 0.004) (Table 4).

**Table 4:** Relative risk of high‐grade cervical disease according to age group

HPV test result	Relative risk of CIN2+ (95% CI)	*P* value
Women 30–34 years old		
HPV16(+) versus HPV16(−)	90.0 (4.00–2023.39)	0.005
HPV18/45(+) versus HPV18/45(−)	(no cases)	
HPV16/18/45(+) versus other HR‐HPV(+)	7.2 (0.59–87.77)	0.122
Women 35–39 years old		
HPV16(+) versus HPV16(−)	1.8 (0.17–19.25)	0.613
HPV18/45(+) versus HPV18/45(−)	2.9 (0.55–14.85)	0.214
HPV16/18/45(+) versus other HR‐HPV(+)	3.2 (0.64–16.38)	0.155
Women 40–44 years old		
HPV16(+) versus HPV16(−)	6.7 (0.68–52.90)	0.107
HPV18/45(+) versus HPV18/45(−)	1.1 (0.10–12.36)	0.923
HPV16/18/45(+) versus other HR‐HPV(+)	5.08 (0.48–54.02)	0.178
Women ≥35 years old		
HPV16(+) versus HPV16(−)	6.2 (0.73–53.4)	0.095
HPV18/45(+) versus HPV18/45(−)	1.0 (0.10–11.09)	0.973
HPV16/18/45(+) versus other HR‐HPV(+)	3.7 (0.52–25.77)	0.192
Women (all ages)		
HPV16(+) versus HPV16(−)	6.5 (2.21–19.64)	0.001
HPV18/45(+) versus HPV18/45(−)	1.4 (0.46–4.09)	0.568
HPV16/18/45(+) versus other HR‐HPV(+)	4.2 (1.57–11.47)	0.004

CIN2+, cervical intraepithelial neoplasia grade 2 or worse; HPV, human papilloma virus; HR‐HPV, high‐risk HPV types; Other HR‐HPV(−), HPV DNA test negative for high‐risk types other than HPV 16/18/45; Other‐HR HPV(+), HPV DNA test positive for high‐risk types other than HPV 16/18/45; HPV 16(+), HPV DNA test positive for HPV 16; HPV 18/45(+), HPV DNA test positive for HPV 18; HPV 16/18/45(+), HPV DNA test positive for HPV 16, HPV 18, and HPV 45.

## Discussion

This study aimed to assess the feasibility of a CC screening program based on vaginal self‐sampling combined with the Xpert HPV assay in a LMIC. Use of the Xpert HPV assay in a screen‐and‐treat context is supported by a recent study conducted in Zambia, which assessed the sensitivity and specificity of the technique 15. We observed that the Xpert HPV assay can be easily installed and managed by caregivers after a short training period. The only facilities required for its use are electricity, and a dry and temperate working area. The process can be interrupted by power outage; an electrical generator can provide a valuable solution to circumvent this issue. Additionally, because the test results were ready in 1 hour, we were able to provide screening, triage and treatment on the same day for those women needing it. This screen‐and‐treat approach was successful, as the majority of women with an indication for treatment were able to be treated in the same‐day session. This strategy allowed us to achieve a very low rate of patients lost to follow‐up (1.1%). Loss to follow‐up is a particular issue in resource‐constrained contexts, where limited facilities are partly responsible for low patient compliance. A long interval between visits, such as that associated with cytology‐based screening, can result in high rates of loss to follow‐up and, in the long term, high incidence of CC 16.

The prevalence of CIN2+ lesions in women positive for HPV 16 and HPV 18/45 was 35.0% and 11.9%, respectively. The relative risk (RR) for CIN2+ in women positive for HPV 16 and/or HPV 18/45 compared with in women positive for other HR‐HPV genotypes was 4.2 (95% CI: 1.6–11.5). Furthermore, HPV 16‐positive women had 6.5 times the risk of CIN2+ compared with women positive for other HR‐HPV genotypes (95% CI: 2.21–19.64). Such results highlight the importance of genotyping as a valuable tool to identify higher‐risk groups among HPV‐positive women and to increase the specificity of HPV testing. Our findings are consistent with those of previous studies that detected HPV 16, 18 and/or 45 in 71.1% of patients with CC 17. These observations suggest the need for a new type of management of women positive for HPV in developing countries. Indeed, the high RR of developing CIN2+ combined with notable drop‐out rates due to low patient compliance in LMIC suggests to opt for immediate treatment of women with HPV 16/18/45. Considering the high morbidity and mortality of CC and the efficacy and acceptability of thermocoagulation treatment 18, 19, the risks‐benefits balance seems to weight in favor of immediate treatment. Conversely, because of their lower RR of CIN2+, women positive for other HR‐HPV types should be triaged by VIA/VILI to minimize the risk of over‐treatment. Overall, this management algorithm could potentially avoid missing most cervical disease and, in the long term, reduce mortality due to CC in low‐resource settings. The extension of such algorithm on a larger scale, however, requires careful consideration and more solid foundations. While HPV testing has recently been approved as a primary screening method in several industrialized countries, studies conducted in low‐income settings also support its role in the reduction in CC incidence and mortality rates after a single screening round 20. Despite the growing knowledge on the subject, appropriate management of HPV‐positive women has yet to be defined and should be supported by a large body of evidence in order to identify the best pathway in terms of cost‐effectiveness, sustainability, and acceptability.

Viral genotyping allowed us to detect a 2% prevalence of HPV 16, a 4.2% prevalence of HPV 18/45, and a 13.9% prevalence of other HR‐HPV types in the study population. The HPV prevalence in our study population was similar to that observed in other studies in Sub‐Saharan Africa 17, 21, 22. Although the HPV prevalence varies widely among different African regions, previous studies have concluded that, while the HPV test's overall performance can vary between different populations, its positive predictive value (PPV) tends to remain constant 23, 24. This aspect is certainly a strength in favor of HPV‐based screening, as it contributes to render it a valuable tool for CC screening.

According to our results, the sensitivity and specificity of VIA/VILI in detecting CIN2+ among HPV positive women were 80% and 44%, respectively. This finding means that although most HPV positive women were VIA/VILI positive, more than half of them did not actually have precancerous lesions and were, therefore, un‐necessarily treated. Indeed, one of the issues of a screen‐and‐treat approach is the risk of overtreatment 25. It is widely known that one of the main limitations of visual inspection methods for CC screening is its subjectivity 26. For this reason, it is recommended to couple different screening methods in order to potentiate their efficacy and minimize the risks of overtreatment. A recent cost‐effectiveness analysis aiming at estimating the cost of comprehensive CC prevention in LMIC has concluded that, despite the limited sensitivity in the detection of CIN2+, the most convenient screening option in low‐resource settings appears to be HPV testing followed by triage of HPV‐positive women with VIA 27. Considering the high loss to follow‐up rates associated to a multiple‐day screening and treatment approach in developing countries, the benefits of a screen‐and‐treat approach seem to outweigh the risks of overtreatment. The choice of thermocoagulation as the first‐line treatment in this screen‐and‐treat campaign is not casual. This procedure is performed on an out‐patient basis, is associated to minor side effects and, unlike excisional procedures, such as Loop Electrosurgical Excision Procedure (LEEP), it has no known adverse effects on pregnancy 18. Furthermore, unlike other ablative procedures, such as cryotherapy, it does not require cumbersome equipment and can easily be managed 28. Although the currently available evidence on the subject is limited, the results of a meta‐analysis on thermocoagulation have shown that this treatment option, which maintains competitive cure rates with other procedures, is particularly suited for CC screen‐and‐treat programs, particularly in low‐resource settings 18.

One strength of our study was the low number of patients lost to follow‐up (1.1%). We observed a drastic reduction in loss to follow‐up, compared with the 25% rate in a previous study of HPV‐positive women in Cameroon 29. Low health literacy, poverty, lack of resources, and geographical conditions are some of the barriers of follow‐up of Cameroonian women. We assume that reduction in loss to follow‐up in our study was a result of the screen‐and‐treat approach which could, in the long term, radically increase program effectiveness. Another strength of our study was the low number of invalid HPV tests. Only 9 tests out of 1012 (0.9%) were invalid. This lower percentage in comparison with previous studies 30 can be explained by the limited delay (from a few minutes to less than 2 hours) between self‐sampling and sample analysis, which was made possible by using a point‐of‐care HPV test. Moreover, women with invalid test results could immediately repeat the procedure and obtain results on the same day. Another strength of our study was the use of dry swabs for the cervicovaginal collection. A recent randomized trial indicated that self‐collected HPV swabs can be successfully transported in a dry state at ambient temperature without altering specimen integrity 31. Because dry swabs are less expensive, do not need refrigeration, and are easy to handle for both participants and healthcare workers compared with wet medium, this approach contributes to making the CC screening approach of this study reproducible in low‐resource settings.

In this study, we did not follow the manufacturer's protocol for sample collection for the Xpert HPV assay, as we used a 0.9% NaCl solution for sample collection instead of the manufacturer's proprietary solution, PreservCyt. This choice reflects a real‐life situation that can be frequently encountered in low‐income settings, where the availability of resources is not always guaranteed. Because NaCl solution is cheaper and readily available in resource‐constrained settings, its use in future screening programs is recommended. The low number of invalid results in our study supports this recommendation. However, equivalence of the two methods has yet to be demonstrated through appropriately designed studies. Our study was limited by the small number of CIN2+ cases in the study population. Estimates based on a larger number of cases would provide more accurate, reliable results. Another limitation of this study is that, although our results allow us to make optimistic assumptions about the management of HPV‐positive women, this pilot study does allow us to draw a conclusion on the subject. Such delicate matter requires careful consideration and should be supported by further, prospective studies.

In conclusion, we have shown that rapid HPV testing is technically accessible and appropriate in low‐resource settings. Our results show that a single‐day, screen‐and‐treat approach can drastically increase compliance with a screening program in a developing country. The success of this type of CC prevention program, however, must further be assessed based on its capacity to ensure sustainability and appropriate scale‐up to the general population of the country and other low‐resource settings.

## Conflicts of Interest

The authors declare no conflict of interest.
